# Comorbidity between Klinefelter syndrome and diaphragmatic hernia. A case report

**DOI:** 10.1590/1516-3180.2014.1325737

**Published:** 2014-07-29

**Authors:** Carolina Melendez Valdez, Stephan Philip Leonhardt Altmayer, Adyr Eduardo Virmond Faria, Aline Weiss, Jorge Alberto Bianchi Telles, Paulo Renato Krall Fell, Luciano Vieira Targa, Paulo Ricardo Gazzola Zen, Rafael Fabiano Machado Rosa

**Affiliations:** I MD. Physician, Gynecology and Obstetrics Program, Hospital Materno Infantil Presidente Vargas (HMIPV), Porto Alegre, Rio Grande do Sul, Brazil; II Undergraduate Medical Student, Universidade Federal de Ciências da Saúde de Porto Alegre (UFCSPA), Porto Alegre, Rio Grande do Sul, Brazil; III MD. Pediatric Surgeon, Hospital Materno Infantil Presidente Vargas (HMIPV), Porto Alegre, Rio Grande do Sul, Brazil; IV MD. Neonatologist, Hospital Materno Infantil Presidente Vargas (HMIPV), Porto Alegre, Rio Grande do Sul, Brazil; V MD. Fetologist, Fet al Medicine, Hospital Materno Infantil Presidente Vargas (HMIPV), Porto Alegre, Rio Grande do Sul, Brazil; VI MD. Obstetrician, Fet al Medicine, Hospital Materno Infantil Presidente Vargas (HMIPV), Porto Alegre, Rio Grande do Sul, Brazil; VII MD. Pediatric Radiologist, Hospital Materno Infantil Presidente Vargas (HMIPV), Porto Alegre, Rio Grande do Sul, Brazil; VIII PhD. Adjunct Professor of Clinical Genetics and of the Postgraduate Program on Pathology, Universidade Federal de Ciências da Saúde de Porto Alegre (UFCSPA), and Clinical Geneticist, Universidade Federal de Ciências da Saúde de Porto Alegre (UFCSPA) and Complexo Hospitalar Santa Casa de Porto Alegre (CHSCPA), Porto Alegre, Rio Grande do Sul, Brazil; IX PhD. Clinical Geneticist, Universidade Federal de Ciências da Saúde de Porto Alegre (UFCSPA), Complexo Hospitalar Santa Casa de Porto Alegre (CHSCPA) and Hospital Materno Infantil Presidente Vargas (HMIPV), Porto Alegre, Rio Grande do Sul, Brazil

**Keywords:** Klinefelter syndrome, Sex chromosomes, Karyotype, Hernia, diaphragmatic, Prenatal diagnosis, Síndrome de Klinefelter, Cromossomos sexuais, Cariótipo, Hérnia diafragmática, Diagnóstico pré-natal

## Abstract

**CONTEXT:**

Intrathoracic cystic lesions have been diagnosed in a wide variety of age groups, and the increasing use of prenatal imaging studies has allowed detection of these defects even in utero.

**CASE REPORT:**

A 17-year-old pregnant woman in her second gestation, at 23 weeks of pregnancy, presented an ultrasound with evidence of a cystic anechoic image in the fet al left hemithorax. A morphological ultrasound examination performed at the hospital found that this cystic image measured 3.7 cm x 2.1 cm x 1.6 cm. Polyhydramnios was also present. At this time, the hypothesis of cystic adenomatoid malformation was raised. Fet al echocardiography showed only a dextroposed heart. Fet al magnetic resonance imaging produced an image compatible with a left diaphragmatic hernia containing the stomach and at least the first and second portions of the duodenum, left lobe of the liver, spleen, small intestine segments and portions of the colon. The stomach was greatly distended and the heart was shifted to the right. There was severe volume reduction of the left lung. Fet al karyotyping showed the chromosomal constitution of 47,XXY, compatible with Klinefelter syndrome. In our review of the literature, we found only one case of association between Klinefelter syndrome and diaphragmatic hernia.

**CONCLUSIONS:**

We believe that the association observed in this case was merely coincidental, since both conditions are relatively common. The chance of both events occurring simultaneously is estimated to be 1 in 1.5 million births.

## Abstract (pt)

**CONTEXTO:**

Lesões císticas intratorácicas são diagnosticadas em ampla variedade de faixas etárias, e o uso aumentado dos estudos de imagem pré-natal tem permitido a detecção desses defeitos ainda intraútero.

**RELATO DO CASO:**

Uma gestante de 17 anos que estava em sua segunda gravidez, com 23 semanas de gestação, apresentava ultrassom com evidência de imagem cística anecoica no hemitórax esquerdo fet al. O ultrassom morfológico realizado no hospital verificou que esta media 3,7 cm x 2,1 cm x 1,6 cm. Evidenciou-se também a presença de polidrâmnio. Neste momento, levantou-se a hipótese de malformação adenomatoide cística. A ecocardiografia fet al mostrou apenas coração desviado para a direita. A ressonância magnética fet al revelou imagem compatível com hérnia diafragmática à esquerda, contendo estômago e, pelo menos, primeira e segunda partes do duodeno, lobo esquerdo do fígado, baço, segmentos de intestino delgado e porções do cólon. O estômago mostrava-se muito distendido e o coração, deslocado para a direita. Havia redução importante do volume do pulmão esquerdo. O cariótipo fet al mostrou constituição cromossômica 47,XXY, compatível com a síndrome de Klinefelter. Em nossa revisão da literatura, encontramos apenas um caso de associação entre síndrome de Klinefelter e hérnia diafragmática.

**CONCLUSÃO:**

Acreditamos que a associação observada neste caso foi puramente uma coincidência, uma vez que ambas as condições são relativamente comuns. A chance de os dois eventos ocorrerem simultaneamente é estimada em 1 em 1,5 milhões de nascimentos.

## INTRODUCTION

Intrathoracic cystic lesions have been diagnosed in a wide variety of age groups, and the increasing use of prenatal imaging studies has allowed detection of these defects even in utero. Diaphragmatic hernias are intrathoracic lesions characterized by a posterolateral defect of the diaphragm that allows passage of the abdominal viscera into the thorax.^1^

Klinefelter syndrome is considered to be the most common disorder of sex chromosomes. It was first described by Harry F. Klinefelter and colleagues in 1942 and it is clinically characterized by features related especially to gonadal development and fertility. Other findings frequently observed include tall stature, delayed speech development, learning disabilities and behavioral problems.^2^ However, Klinefelter syndrome may be difficult to diagnose without karyotyping analysis, especially in the fetus during pregnancy and during childhood, because the main features of the syndrome, such as azoospermia and increased gonadotropin levels, are observed only after the puberty period.^2^^,^ 3

Our aim was to report on a rare case of association between Klinefelter syndrome and diaphragmatic hernia, with diagnosis in utero. 

## CASE REPORT

A 17-year-old pregnant woman in her second gestation, with a prior history of a pregnancy loss, presented a nuchal translucency measurement of 2 mm, at the first-trimester screening. Obstetric ultrasound revealed the presence of a cystic anechoic image in the left hemithorax of the fetus. On average, she smoked five cigarettes per day. She denied using illicit drugs or alcohol. Her husband was a healthy and non-consanguineous 19-year-old man. There was no history of malformations or genetic diseases in the family.

**Figure 1:**
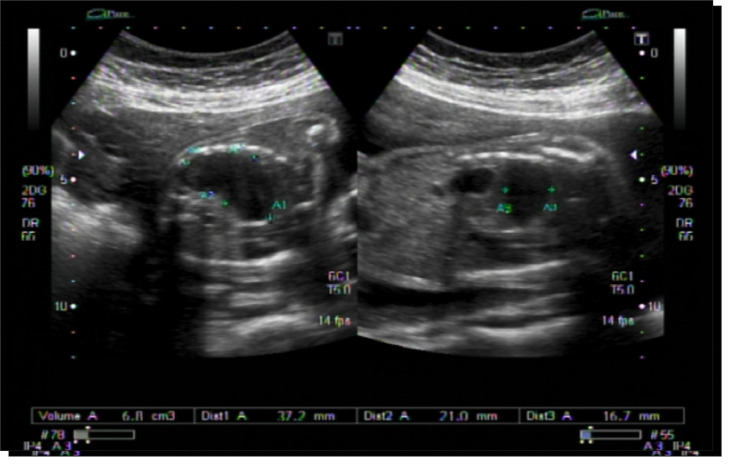
Fet al ultrasound showing the intrathoracic cystic lesion.

A morphological ultrasound examination performed at the hospital, at 23 weeks and 6 days, confirmed the finding of the fet al cystic image. It measured 3.7 cm x 2.1 cm x 1.6 cm. Polyhydramnios was also present (Figure 1). Cystic adenomatoid malformation was initially considered as a diagnosis for the patient. Fet al echocardiography only showed a dextroposed heart. 

**Figure 2:**
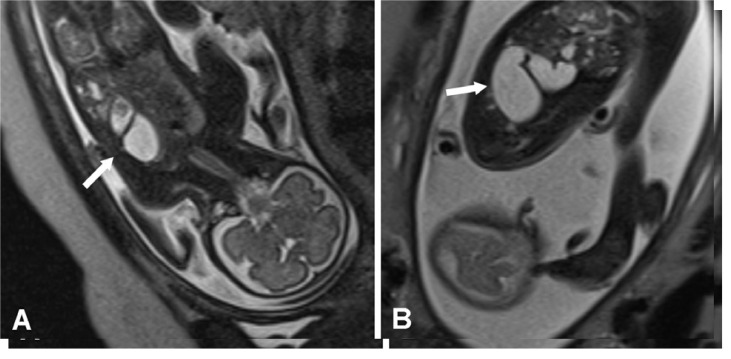
Fet al magnetic resonance imaging showing findings compatible with left-side diaphragmatic hernia (see arrows).

Fet al magnetic resonance imaging showed polyhydramnios and findings compatible with left diaphragmatic hernia involving the stomach and at least the first and second portions of the duodenum (distended with fluid), left lobe of the liver, spleen, small intestine segments and portions of the colon. The stomach was greatly distended and the heart was shifted to the right. There was severe volume reduction of the left lung (Figure 2). Fet al karyotyping showed that the chromosomal constitution was 47,XXY, which was compatible with Klinefelter syndrome. 

The child was born through cesarean section, at 34 weeks of gestation, with weight of 2,070 g, length of 45 cm, head circumference of 31 cm and Apgar scores of 6 at the first minute and 8 at the fifth minute. No dysmorphic features were seen in the child. He did not present micropenis or cryptorchidism. He underwent surgery on the diaphragmatic hernia on the fifth day of life. Duodenal atresia was also verified. An echocardiography showed the presence of an atrial septal defect of ostium secundum type. The child died a few days later due to complications from pulmonary hypoplasia.

## DISCUSSION

In our review of the literature, we found only one case of an association between Klinefelter syndrome and diaphragmatic hernia (Table 1).^4 ^The etiology of the diaphragmatic hernia is largely unknown and most cases are isolated, i.e. not associated with other malformations or conditions. However, it may be a component of some syndromes, such as Pallister Killian, Fryns and Brachman-De Lange.1 We believe that the association observed in the present case was merely coincidental, since both conditions are relatively common. The frequency of diaphragmatic hernia has been postulated to be up to 5 in 10,000 births, and about half of the patients are male.^1^ The incidence of Klinefelter syndrome is around 1 in 660 among newborn boys,^5^ and thus the estimate for occurrences of both events together would be around 1 in 1.5 million births. This chance is similar to that described by Taheri and Kadir4 for a fetus to be affected by both conditions.

**Table 1:** Results obtained from each database using the descriptors corresponding to the main features presented by the fetus/patient. The search in these databases was conducted on June 26, 2013.

Database	Search strategy	Results	
		Found	Related
Medline (Medical Literature Analysis and Retrieval System Online; (via PubMed)	"Klinefelter syndrome" OR "47,XXY" AND "Hernia, Diaphragmatic"	1	1 case report^4^
Embase (Excerpta Medica Database; via Elsevier)	"Klinefelter syndrome" OR "47,XXY" AND "Hernia, Diaphragmatic"	39	0
Lilacs (Literatura Latino-Americana e do Caribe em Ciências da Saúde; via Biblioteca Virtual em Saúde)	"Klinefelter syndrome" OR "47,XXY" AND "Hernia, Diaphragmatic"	0	0
SciELO (Scientific Electronic Library Online)	"Klinefelter syndrome" OR "47,XXY" AND "Hernia, Diaphragmatic"	0	0

Samangaya et al.^6^ reported that the risk of having a chromosomal abnormality in a case of congenital diaphragmatic hernia after being diagnosed through ultrasound is up to 15.9%, which enhances the importance of fet al karyotyping in this situation.^7^ The chromosomal abnormalities observed among patients with congenital diaphragmatic hernia include tetrasomy 12p mosaicism and trisomy 18.^1^^,^ 8 Interestingly, cystic adenomatoid malformation was our first hypothesis for the intrathoracic cystic lesion observed in the fetus, and this has been poorly associated with chromosomal abnormalities, especially as an isolated defect.^7^

The prognosis for diaphragmatic hernia is still very poor.^6^ Fetuses with Klinefelter syndrome usually do not present associated major malformations and, differently from other chromosomal anomalies, such as Turner syndrome or trisomy 13 and 18, do not show increased rates of intrauterine mortality.^2^^,^ 9 Although the risk of dying due to a variety of diseases, such as malignant neoplasms, diabetes type 2 and respiratory and circulatory system diseases may be greater among Klinefelter patients,^10 ^we believe that the chromosomal anomaly present in our patient did not interfere with the prognosis associated with his diaphragmatic hernia.

## CONCLUSIONS

We believe that the association observed in this case was merely coincidental, since both conditions are relatively common. Further reports would be needed in order to confirm a possible association between Klinefelter syndrome and diaphragmatic hernia. Our report also highlights the importance of using magnetic resonance imaging for elucidating fet al intrathoracic cystic lesions.
